# Social, cultural, and land use determinants of the health and well-being of Aboriginal peoples of Canada: A path analysis

**DOI:** 10.1057/jphp.2013.27

**Published:** 2013-06-13

**Authors:** Shashi Kant, Ilan Vertinsky, Bin Zheng, Peggy M Smith

**Affiliations:** a University of Toronto, 33 Willcocks Street, Toronto, Ontario, Canada M5S 3B3; b Sauder School of Business, University of British Columbia, 2053 Main Mall, Vancouver, BC, Canada V6T 1Z2; c Lakehead University, 955 Oliver Road, Thunder Bay, Ontario, Canada P7B 5E1

**Keywords:** Aboriginal, Canada, culture, First Nations, holistic

## Abstract

We explored the contributions of social, cultural, and land use (SCLU) factors to Aboriginal well-being and health using path analysis and data collected from 2 of 614 First Nations in Canada. Information gathered from a structured questionnaire with questions related to seven domains of well-being and contributing factors led to key findings: (i) the SCLU domain is the most important; (ii) the most important SCLU factors are the percentage of household meals of traditional diets and the impact of government regulations on land use; (iii) the most important Health domain factors are the prevalence of mental and psychological problems and the quality of health services; and (iv) the SCLU factors of access to cultural sites, the freedom to participate in spiritual activities, and the impact of government regulations on social and cultural life have a profound effect on mental health. Improving Aboriginal well-being and health may depend on incorporating SCLU factors into new, holistic policies.

## Introduction

The indigenous peoples of North America and their descendants are known as Aboriginal peoples. The Canadian Constitution recognizes three groups of Aboriginal peoples: Indians (commonly referred as First Nations), Inuit (indigenous peoples inhabiting the Arctic region), and Métis (descendant of mixed heritage of First Nations and Europeans). The 2006 census of Canada found that Aboriginal peoples of Canada (∼1.17 million) were 3.8 per cent of the Canadian population, and ∼0.3 per cent of the World's Indigenous populations (more than 370 million) living in 90 countries.^1^ Approximately 60 per cent of Canadian Aboriginal peoples belong to First Nations, and 57 per cent First Nations people live in 614 First Nations communities (also called ‘reserves') and the other 43 per cent in urban areas.^2^ A ‘reserve' is government-owned land set aside by the Canadian government for the use of a First Nation's people.

If the Aboriginal peoples of Canada were considered as a separate national entity, that nation would have ranked 48th out of 175 countries in the United Nations' Human Development Report, while Canada regularly ranks at or near the top.^3^ Colonization, infringement of Aboriginal land rights, and the residential school system have had a significant adverse impact on Aboriginal peoples,^4, 5^ resulting in health disparities and ‘trapping' them in an ‘endless circle of disadvantage'.^6, 7^

Analysis of the determinants of Aboriginal well-being and health is incomplete without an understanding of the Aboriginal worldview.^8, 9^ Collectivism, non-possession, harmony with nature, and seeing all things as interconnected are key features of the Aboriginal worldview.^10^ Holistic health is also a key ingredient of the Aboriginal health system.^5, 10^ In this system, social, cultural, and land use (SCLU) factors are the essential foundation of Aboriginal well-being and health.^11, 12, 13^

Given that the Aboriginal worldview was suppressed,^14^ the search for solutions to better Aboriginal health, by the Canadian public health policy and health systems, centers on Western medicine, whereas the search for solutions for well-being focuses on economic growth. Arguably, more effective solutions may lie outside these boundaries and could be derived from a better understanding of the SCLU determinants of Aboriginal health and well-being.^12^ Many studies using cultural and value propositions have discussed the role of these determinants,^15, 16, 17^ but there is no evidence-based study of Aboriginal well-being and health using SCLU determinants and the holistic approach.

Our article begins to address this knowledge gap with a first exploratory and evidence-based study of First Nations peoples living on ‘reserves' – the largest group among Aboriginal people. The three groups of Aboriginal peoples have distinct histories, languages, and cultural practices, but land-based cultures and the *Mother Earth* as a source of everything that we need for survival are common features of all Aboriginal peoples. Our study may be a good start, but further studies will be required before applying our findings more widely.

Researchers widely accept that the concept of well-being is multidimensional and encompasses all important domains of life.^18, 19^ Use of arbitrarily chosen objective measures of well-being to guide public policy, such as income and employment, may impose policymakers' values on peoples with different values. Subjective measures that give voice to affected populations may help create more effective and responsive policies. We use judgments by Aboriginal peoples about their perceived distances from attaining their goals and aspirations in each of the domains they identify as important to their well-being, as well as their assessment of satisfaction with life as a whole.^20, 21, 22^ We use path analysis,^23^ a technique useful for complex and holistic modeling, to impute the interrelationships between levels of satisfaction within a variety of life domains, as well as the direct and indirect contributions that this satisfaction within each domain makes to the satisfaction with life as a whole. We conclude with a discussion on policy interventions.

## Methods

### Design and data collection

**Figure 1:**
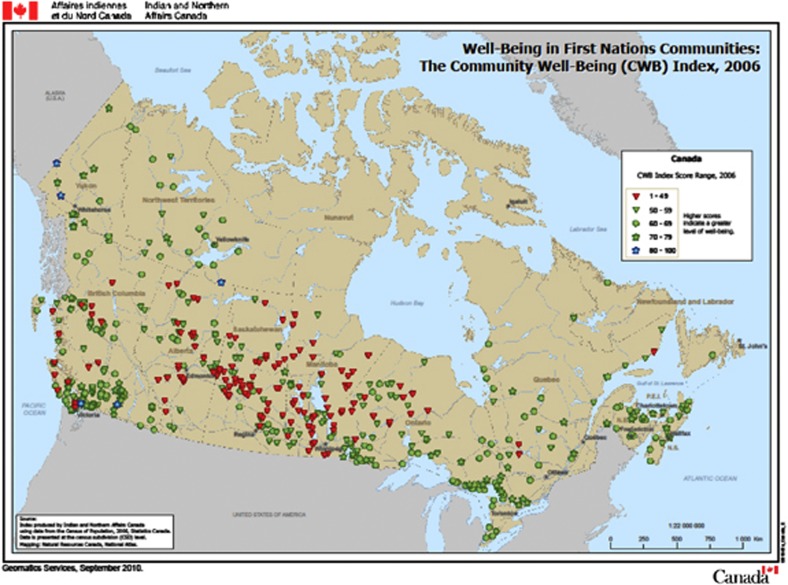
Well-being in First Nations of Canada. *Source*: This map is a copy of the version available at: http://www.aadnc-aandc.gc.ca/DAM/DAM-INTER-HQ/STAGING/texte-text/ai_rs_pubs_cwb_map_canada_1321640456592_eng.pdf, accessed 1 May 2013.

The study team conducted this research in two First Nations – one in the province of Ontario (population 600, 120 households) and other in British Columbia (population 1500, 275 households). In 2006, the Community Well-being (CWB) Indices for the Ontario and British Columbia communities in our study were 58 and 75, respectively, as compared with the average of 57 for all First Nations.^24^ These two nations represent an above average standard of living among the First Nations. The geographical distribution of the CBW Indices of all First Nations appears in Figure 1. Unsafe drinking water, crowded homes, high unemployment, high rates of suicide, diabetes, tuberculosis, and limited access to quality health care are dominant features of First Nations. The two nations under study are not identified in Figure 1 because of our commitment of confidentiality to them.

The two First Nations participated as supporting partners in the research project actively providing feedback to shape its design. Once funding was granted, we obtained formal approval to conduct the study from the Research Ethics Board of the University of Toronto and the Chief's representatives of each First Nation. To design a questionnaire, we held in each First Nation focus groups with the heads of households and one-to-one discussions with Elders as a basis for identifying key domains that characterize their well-being and factors that influence these domains. We designed a preliminary questionnaire based on these discussions plus theoretical perspectives. We finalized it based on further inputs from Elders, elected representatives, and the results of pre-testing. The Chief's office in each Nation facilitated these processes.

The Chief's office informed all households about the study. We trained a community member from each First Nation to facilitate data collection, and then a researcher and the community member delivered questionnaires in person to 355 households where an adult was available. The community member helped respondents, as required, in understanding questions. We collected 316 questionnaires (314 with complete information; a response rate of 90 per cent). We submitted the first draft of this article to the Chiefs' offices. We addressed their suggestions. We are working closely with these First Nations for wider dissemination of our findings.

### Instrument

The questionnaire included questions about satisfaction with: (i) general well-being; (ii) six domains of well-being, similar to Cummins^25^ and Argyle^26^ – Education, Employment, Health, Housing, Income, and Social–Cultural; and (iii) a seventh domain of Land Use, identified by Aboriginal peoples. For assessing satisfaction, we asked respondents to rank on a 7-point Likert scale the level of satisfaction for their household for a 1-year period preceding the survey (1=extremely unsatisfied and 7=extremely satisfied). The questionnaire also included questions related to influencing factors for each domain. The details of influencing factors and their measurements appear in Table 1.

### Data analysis

We analyzed these data using path analysis with IBM SPSS Amos 19.0 software,^23,27^ employing the Maximum likelihood method of estimation. Given the limitations of *χ*^2^ statistics,^28^ we also used the following fit measures: Root Mean Square Error of Approximation (RMSEA), Comparative Fit Index (CFI), Goodness-of-Fit Index (GFI), and Normed Fit Index (NFI).

### Results of Path Analysis Models

Preliminary analysis of the data showed satisfaction levels from the social–cultural and the land use domains to be highly correlated. Thus, we combined these two domains to form the SCLU *domain*. The average levels of general satisfaction, the SCLU domain satisfaction, and the Health domain satisfaction for the BC First Nation were 5.02, 4.44, and 5.10, respectively, whereas these satisfactions for the Ontario First Nation were 5.30, 4.62, and 5.32, respectively.

### Results of general well-being model

**Figure 2:**
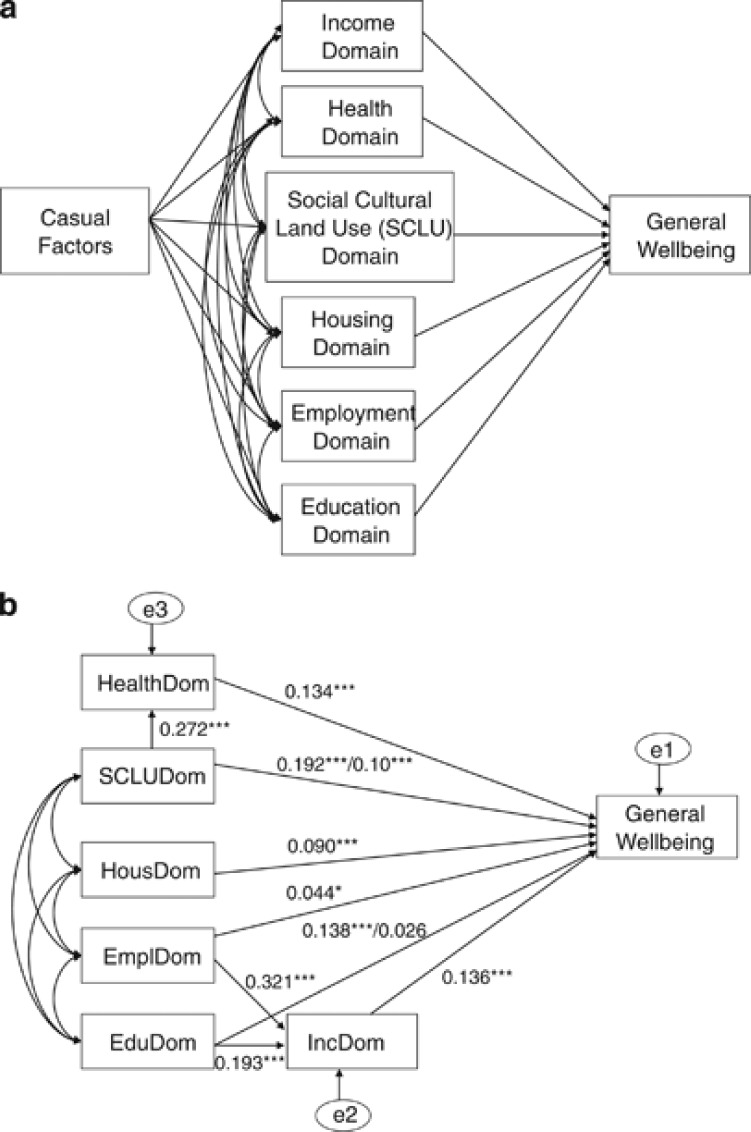
(**a**) Path diagram of multi-domain Aboriginal well-being; (**b**) Path coefficients of general well-being of Aboriginal peoples (female versus male). *Note*: A single number for the path coefficient means that females and males have the same path coefficient. In the case of two numbers, the first number indicates the path coefficient for females and the second number for males. Error terms are represented by e1, e2, and e3.

We present our hypothesized model of general well-being in Figure 2a. In this figure, all hypotheses related to the contribution of a determining factor to general well-being are shown by one-way arrows; hypotheses related to correlations between two variables are shown by two-way arrows. We tested this model separately with data from each First Nation and then with both. Model estimation involved two steps.^29^ First, we tested for all factors of the first order with all possible correlations as data. Second, we tested the model for all factors of the second order. After each trial, we trimmed out of the Model one or more paths, keeping those with path coefficients significant at the 10 per cent level in two of the three tests (tests with BC, Ontario, and with all data from the two). The process produced a model that was robust across the two regions. Test results (*χ*^2^=17.17, dof=17) indicate no significant difference between the BC and Ontario models, thus we based the final model on all data. Test results (*χ*^2^=65.27, dof=7) do suggest differences between female and male models. Further *χ*^2^ tests suggest that the path coefficient for the Education and SCLU domains are significantly different for females and males.

Overall, the ‘goodness-of-fit' indices support the general well-being as well as the Health and the SCLU domain models. Although *χ*^2^ values for these models were significant (*P*=0.00), the values of all other fit indices (RMSEA well below the cut-off limit of 0.10, and NFI, CFI, and GFI close to 0.90) fall within acceptable limits.

The path coefficients of the final general well-being model appear in Figure 2b and the direct, indirect, and total effects of domains in Table 2.

Overall, the model has a good explanatory power and most of the path coefficients are significant at the 1 per cent level. The SCLU domain contributed most to general well-being with the standardized coefficients of the total effect being 0.317 and 0.219 for females and males, respectively. The SCLU domain also influenced general satisfaction indirectly through the Health domain, which makes the total effect (unstandardized) of 0.228 for females and 0.136 for males, respectively.

The unstandardized path coefficient of the Health domain to general satisfaction was 0.134, almost equal to the path coefficient of the Income domain (0.136), and much larger than the path coefficients of the Employment (0.044) and Housing domains (0.090). The path coefficient of the Education domain for females was 0.138 and for males was 0.026, respectively. Thus, for females the Health and Education domains were almost equally important, whereas for males the Health domain was much more important.

### Results of SCLU domain model

A *χ*^2^ value of 13.086 at 5 dof suggests a significant difference between female and male SCLU models. With further tests, we found that the path coefficients of Trappers to TrapInc and TradDiet to TrapInc were significantly different for males and females. Results for the final model appear in Table 2.

TrapInc, Accecultu, LawLandus, and SocTies had a direct influence on the SCLU domain satisfaction, and the path coefficients for these four factors are the same for females and males. Trappers and TradDiets had direct and indirect impacts on the SCLU domain, and the coefficients were different for male and female groups. On the basis of the standardized total effect, TradDiets and LawLandus contributed most to SCLU domain satisfaction. Further, TradDiets influenced SCLU satisfaction indirectly through the income attributable to trapping. TradDiets is also significantly related to TrapInc.

The number of trappers in a household had a negative path coefficient to SCLU satisfaction, possibly because of decreasing returns from trapping or decreasing availability of labor for other activities of this domain. The number of trappers influenced traditional diets and trapping income, and thus influenced indirectly SCLU satisfaction. The total effect of Trappers to SCLU satisfaction was positive, and the unstandardized total effect coefficient was larger for females than males. The influence on SCLU satisfaction of the perceived impact of government regulations on land use was larger than the impact of social ties and household access to Aboriginal cultural sites.

### Results of health domain model

A *χ*^2^ test suggested that significant differences exist between female and male domain models (*χ*^2^=10.148, dof=4). The results for the final model appear in Table 2. A preliminary model of this domain included income, education, employment, and age as additional influencing variables, but these were not found to be significant and were deleted in the final model.

The physical health factors (ExtOrgSev and IntOrgSev) and mental health factors (MentalFreq and MentalOc) had, as expected, negative direct impacts, whereas the level of health services (HealthServ) had a positive direct impact on the satisfaction of both males and females with the Health domain. The impact of frequent occurrences of mental and psychological problems had a higher impact on Health satisfaction than experiencing severe internal or external organ illnesses. Female satisfaction was affected more than that of males when experiencing occasional mental and psychological problems. Access to cultural activities reduced the frequency of mental and psychological problems, and contributed indirectly to health satisfaction. A positive contribution of government regulations on cultural and social life reduced the incidences of occasional mental problems, and the impact was higher for females compared with males. Satisfaction with health services had a positive impact on satisfaction with the health domain.

### Limitations, Conclusions, and Policy Implications

Our results provide the first strong empirical evidence of the Aboriginal worldview of a holistic approach to well-being and health, and strong influence of SCLU factors. Our study is exploratory and based on a very small sample of First Nations peoples living on-reserves and belonging to the upper-half of First Nations on the CWB ranking. Our findings, therefore, cannot be generalized to all First Nations peoples, specifically people living off-reserve, or to Inuit and Metis peoples. On the other hand, this study of two First Nations in geographically distinct regions of Canada with statistically significant differences in their standard of living found no statistically significant differences in the models derived for each, suggesting the robustness of our measurements and estimations. It is also quite plausible that the importance of SCLU factors to the general well-being of the lower-half of First Nations could be much higher than that of the upper-half group. If so, the key findings, summarized below, can be treated as a starting point in designing new Canadian policy interventions to improve well-being and health of on-reserve Aboriginal peoples.

### Conclusions and policy implications

First, the SCLU domain, as the most important one for Aboriginal well-being, indicates that income alone is not a good proxy of well-being, as it does not reflect some critical things important to Aboriginal peoples.^30^ This suggests that Aboriginal well-being policies can be improved by recognition of the importance of SCLU domain. As the contributions of the Income and the Health domains are almost equal, Aboriginal well-being policies that place the same importance on Health and Income domains are likely to be better than current ones. For females, the contribution of the Education domain was more than that of the Income and Health domains, but less than that of the SCLU domain. For First Nation women, satisfaction with life seemed dependent on securing the future of their families. Education may support their families to secure both income and health. The relative importance of each domain, however, is not independent of the other domains, and therefore increasing satisfaction in one domain may tend to increase the general satisfaction directly or indirectly through its influence on other domains. Taking advantage of these direct and indirect relationships among domains, in designing Aboriginal well-being policies, may improve Aboriginal well-being outcomes.

Second, the most important contributors to the SCLU domain were the percentage of household meals from traditional diets and the impact of government regulations (law) on land use activities. These results provide analytical support to First Nation perspectives, and suggest that policymakers have an opportunity for positive impact on Aboriginal well-being by developing policies and laws that support these Aboriginal activities. The next two most important factors contributing to the SCLU domain were the sense of belonging to a local community and social ties and access to First Nation cultural sites, implying that policies that protect and facilitate access to Aboriginal cultural sites and the fabric of community belongingness may also contribute to Aboriginal well-being.

Third, in the Health domain, frequent occurrences of mental and psychological problems for males and both occasional and frequent occurrences of mental and psychological problems for females are critical factors. Development of national Aboriginal mental health policy and culturally appropriate mental health programs that include traditional healing practices are likely to be critical for Aboriginal health. Improved access to cultural sites and freedom to participate in spiritual activities are likely to reduce prevalence of mental and psychological problems. Governments can reduce prevalence of mental and psychological problems by developing regulatory systems that support social and cultural life. These might also contribute to health satisfaction by investing in health services improvement. Provision of health services, however, should be only one component in a comprehensive health and well-being policy that is sensitive to the role of SCLU factors and their contributions.

Finally, SCLU factors are deeply embedded determinants of Aboriginal health and welfare,^17^ and our results provide strong empirical evidence for likely benefits of repositioning Aboriginal health and welfare policies from their current Western science-based perspective to an integrated knowledge perspective. Public policymakers can also learn from case studies of First Nations, Inuit, and Metis communities that have retained traditional health knowledge and practices grounded in SCLU determinants. Such case studies may help refine policies by developing more nuanced approaches that respond more sensitively to variations in SCLU context. Future studies focused on a wider spectrum of Aboriginal peoples are needed to validate, modify, and extend our findings and policy recommendations.

## Figures and Tables

**Table 1:** Explanation and measurement of influencing factors

*Abbreviation*	*Explanation and measurement*
AcceCultu	Access to First Nation cultural sites (1-4)^a^
ExtOrgFreq	Frequent occurrence of external organ illnesses in household (Yes/No)
HealServ	Satisfaction with government health services (1-4)^a^
IntOrgFreq	Frequent occurrence of internal organ illnesses in household (Yes/No)
Lawlanduse	Impact of government law on household First Nation land use activities (1-4)^a^
LawCultu	Impact of government law on household First Nation cultural and social life (1-4)^a^
MentalFreq	Frequent occurrence of mental and psychological problems in household (Yes/No)
MentalOc	Occasional occurrence of mental and psychological problems in household (Yes/No)
SocTies	Sense of belonging to local community and social ties (1-4)^a^
Spiritual	Household freedom to participate in First Nation spiritual activities (1-4)^a^
TradDiets	Percentage of typical household meal that comes from First Nation traditional diets (that is, diets obtained from First Nation land use activities such as hunting, fishing, gathering, and so on)
TrapInc	The percentage of income attributable to trapping
Trappers	Number of trappers in household

a 4-point Likert scales (1=very low, 4=very high).

**Table 2:** Gender-wise direct, indirect, and total effect of factors in different models

*Path*	*Path coefficient female/male*	*Total effect female/male*	*Standard path coefficient female/male*	*Standard total effect female/male*
*Effects of different domains on the general well-being of First Nation people*				
IncDom←EmplDom	0.321*	0.321	0.448	0.448
IncDom←EduDom	0.193*	0.193	0.254	0.254
HealthDom←SCLUDom	0.272*	0.272	0.315	0.315
Well-being←HealthDom	0.134*	0.134	0.161	0.161
Well-being←EmplDom	0.044***	0.087	0.086	0.173
Well-being←IncDom	0.136*	0.136	0.193	0.193
Well-being←HousDom	0.090*	0.090	0.158	0.158
Well-being←EduDom	0.138*/0.026	0.164/0.052	0.257/0.043	0.306/0.087
Well-being←SCLUDom	0.192*/0.10*	0.228/0.136	0.267/0.161	0.317/0.219
				
*Effects of different factors on the satisfaction of SCLU domain*				
TradDiets ←Trappers	6.365*	6.265	0.362	0.362
TrapInc←Trappers	9.764*/6.808*	11.299/7.461	0.661/0.485	0.765/0.532
TrapInc←TradDiets	0.241*/0.103**	0.241/0.103	0.278/0.155	0.278/0.155
SCLUDom←Trappers	−0.203**	0.085/0.046	−0.121	0.051/0.035
SCLUDom←TradDiets	0.027*	0.030/0.028	0.285	0.311/0.461
SCLUDom←TrapInc	0.010***	0.010	0.090	0.090
SCLUDom←AcceCultu	0.102**	0.102	0.116	0.116
SCLUDom←LawLanUs	0.295*	0.295	0.291	0.291
SCLUDom←SocTies	0.209*	0.209	0.191	0.191
				
*Effects of different factors on the satisfaction of health domain*				
MentalOc←LawCultu	−0.033***/−0.010	−0.033/−0.010	−0.091/−0.026	−0.091/−0.026
MentalOc←Spiritual	−0.040/−0.172*	−0.040/−0.172	−0.118/−0.483	−0.118/−0.483
HealServ←MentalOc	−0.155***	−0.155	−0.098	−0.098
MentalFreq←AcceCultu	−0.022**	−0.022	−0.122	−0.122
HealthDom ←ExtOrgFreq	−0.500*	−0.500	−0.151	−0.151
HealthDom←MentalFreq	−1.153*	−1.153	−0.248	−0.248
HealthDom←MentalOc	−0.507*/−0.104	−0.564/−0.161	−0.220/−0.044	−0.244/−0.068
HealthDom←HealServ	0.368*	0.368	0.252	0.252
HealthDom←IntOrgFreq	−0.477*	−0.447	−0.128	−0.128
HealServ←Spiritual	−0.006/0.027	−0.006/0.027	−0.012/0.053	−0.012/0.053
HealthDom←Spiritual	−0.022/0.028	−0.022/0.028	−0.029/0.033	−0.029/0.033
HealServ←LawCultu	0.005/0.001	0.005/0.001	0.009/0.003	0.009/0.003
HealthDom←LawCultu	0.019/0.002	0.019/0.002	0.022/0.002	0.022/0.002
HealthDom←AcceCultu	0.025/0.025	0.025/0.025	0.030/0.028	0.030/0.028

*Notes*: * Significant at 1 per cent significance level; ** significant at 5 per cent significance level, and *** significant at 10 per cent significance level. Significance levels are shown for path coefficients, and the same levels of significance are applicable for standard path coefficients. Total effect and standard total effect are calculated from different path coefficients, and therefore level of significance is not relevant.
